# *Candida* spp./Bacteria Mixed Biofilms

**DOI:** 10.3390/jof6010005

**Published:** 2019-12-20

**Authors:** Maria Elisa Rodrigues, Fernanda Gomes, Célia F. Rodrigues

**Affiliations:** 1 CEB, Centre of Biological Engineering, LIBRO–Laboratório de Investigação em Biofilmes Rosário Oliveira, University of Minho, 4710-057 Braga, Portugal; elisarodrigues@deb.uminho.pt (M.E.R.); fernandaisabel@deb.uminho.pt (F.G.); 2 LEPABE–Dep. of Chemical Engineering, Faculty of Engineering, University of Porto, 4200-465 Porto, Portugal

**Keywords:** *Candida* spp., bacteria, biofilm, antimicrobial resistance, fungal–bacteria interaction

## Abstract

The ability to form biofilms is a common feature of microorganisms, such as bacteria or fungi. These consortiums can colonize a variety of surfaces, such as host tissues, dentures, and catheters, resulting in infections highly resistant to drugs, when compared with their planktonic counterparts. This refractory effect is particularly critical in polymicrobial biofilms involving both fungi and bacteria. This review emphasizes *Candida* spp.-bacteria biofilms, the epidemiology of this community, the challenges in the eradication of such biofilms, and the most relevant treatments.

## 1. An Overview of Single and Polymicrobial Biofilms Involving *Candida* spp. and Bacterial Species

Microorganisms can naturally accumulate on a wide variety of surfaces where they form sessile communities, as mono or polymicrobial biofilms. Household/industrial, biomaterials and/or biological surfaces are some of the substrates that can be colonized by microorganisms [1]. Indeed, the ability to form biofilms is an important virulence factor for pathogenic microorganisms. It is defined as complex and dynamic microbial 3D structures, consisting of attached cells encased in a self-synthesized matrix of extracellular polymeric substances (EPSs) [2]. Sessile cells are protected from the surrounding environment by the extracellular matrix that covers the cell and is therefore a key factor of drug resistance [3,4]. Biofilm-associated infections are more difficult to treat and control since sessile cells are 10- to 1000-fold more resistant than their planktonic counterparts [5]. This mode of growth confers some advantages to its members, including exchange of substrate, resistance to antimicrobial drugs, immune system, mechanical and environmental stresses, adhesion ability, nutritional sources, and cellular communication [6]. Accordingly, cellular structures enable microbial communication by *quorum sensing* and their adaptation to several stressful conditions and presents a propensity to cause diseases. Due to their “adaptative” resistance, once established, bacterial and fungal biofilm-associated infections are very hard to treat and eradicate. 

A typical microbial biofilm formation involves several steps namely the attachment to biotic or abiotic surfaces (formation of micro-colonies/development of young biofilm), maturation (differentiation of structured mature biofilm), and detachment (dispersal of mature biofilm) [7]. Thus, biofilms can serve as a reservoir for pathogenic cells and their release can cause septicemia and evolve into invasive systemic infections of organs and tissues. External factors contribute and influence the biofilm’s characteristics. Among them, the surface where the biofilm is growing, the nutrients available, and the inhibitors present in the surrounding environment or the presence of antagonistic microorganisms are the most relevant [8]. 

Depending on the situation, bacterial biofilms can be either beneficial or problematic [9]. Still, bacterial biofilms are usually pathogenic and responsible for several diseases, such as nosocomial infections [10]. Among the bacteria responsible for this kind of infection, the majority are bacteria belonging to the known ESKAPE group, namely *Enterococcus faecium*, *Staphylococcus aureus*, *Klebsiella pneumoniae*, *Acinetobacter baumannii*, *Psedomonas aeruginosa*, and *Enterobacter* spp. [11]. Infections derived from these bacteria are nowadays known as being extremely difficult to handle, mostly due to their biofilm formation ability. Additionally, these microorganisms can be found in different contexts, including environmental, industrial, and clinical, which aggravates the possibility of infection. The progress of medical science and the widespread use of medical devices and artificial organs have given rise to the emergence of bacterial biofilm infections [7,12]. Actually, it has been reported that the majority of devices with medical applications may result in biofilm infections, with 65% of all bacterial infections being related to bacterial biofilms [13,14].

Due to the heterogeneity of microorganisms present in the human flora, biofilms that can be generated are mostly polymicrobial, involving either species of the same genus or species from different kingdoms (cross-kingdom microorganisms, such as bacteria and fungi) [15]. Polymicrobial biofilms are now recognized as having higher complicated management [16]. In fact, the diversity, complexity, and the different pathogens associated with polymicrobial biofilms can significantly contribute to severe clinical implications [16]. The interspecies interactions exhibited by polymicrobial biofilms are relevant to the colonization, host response, drug resistance, and disease progression [17,18]. Therefore, all contributions on several aspects behind biofilm formation (e.g., mechanisms of adhesion and signaling involved in multispecies interaction) and antimicrobial resistance are crucial for development of new strategies in the treatment and prevention of polymicrobial biofilms’ infections. The impairment of microbial adhesion and biofilm formation are crucial steps and targets of therapeutic strategies aimed at the inhibition and development of polymicrobial diseases. Correspondingly, the co-aggregation occurring during the polymicrobial biofilm formation is well studied for common pathogens. *Candida* spp., namely *Candida albicans*, was shown to easily form biofilms in combination with other microorganisms, namely bacteria and other yeasts. As an example, *C. albicans* was shown to co-habit with strains, such as *Staphylococcus aureus*, *Streptococcus mutans*, and *Fusobacterium* spp. [19], but more examples of polymicrobial biofilms will be given in the next sections.

Approximately 80% of microbial infections are associated with biofilms, exhibiting high mortality rates [20]. Both yeast and bacteria are able to adhere to biotic or abiotic surfaces, developing into those highly organized communities, which is their preferred mode of growth. Therefore, biofilms formed by yeasts and/or bacteria, and consequently their associated infections, have become increasingly important. Moreover, bacteria and fungi of the *Candida* genus are often found in multispecies biofilms *in vivo* [21], with fungal–bacterial interactions research on the rise. Multi-species biofilms can display different behaviors, namely mutually beneficial (co-aggregation), competitive, and antagonistic interactions. An example of a beneficial interaction between species that live in the same biofilm is the interaction of bacteria and *C. albicans* in the case of oral biofilms. On the other hand, a competitive and antagonistic interaction in multispecies biofilms is observed in the case of *C. albicans* and *Pseudomonas aeruginosa* [21]. 

This review highlights important aspects related to *Candida* spp./bacterial mixed biofilms, namely their epidemiology, microbial resistance, and recent advances in the management of this kind of consortia. Moreover, the challenges found in the development of effective therapies against polymicrobial biofilms are also addressed herein.

## 2. Epidemiology of *Candida* spp./Bacteria Single and Mixed Biofilms

Biofilms’ infections caused by a single microbial species or by a mixture of bacterial and fungal species have increased significantly, contributing to high levels of morbidity and mortality. Indeed, the presence of both eukaryotic and prokaryotic pathogens makes the infections difficult to diagnose as well as to treat, requiring complex multi-drug treatment strategies [17]. Antimicrobials directed towards one species in a mixed-species biofilm often facilitate non-targeted organisms to thrive and continue the infection [22]. In this sense, mixed biofilms represent an understudied and clinically relevant health problem, with the potential to serve as an infectious reservoir for a variety of microorganisms, including bacteria and fungi [17].

### 2.1. Epidemiology of Candida spp. Single Biofilms

*Candida* spp. are major human fungal pathogens, which cause both mucosal and deep tissue infections. The ability of these yeasts to form biofilms on medical devices has a profound effect on its capacity to cause human disease [23]. Infection occurs in 60% of these cases, with *Candida* spp. being responsible for up to 20% of these [24] and mortality rates as high as 30% [25,26,27]. *Candida albicans* can form biofilms on almost any medical device [27], including vascular and urinary catheters, joint prostheses, cardiac valves, artificial vascular bypass devices, pacemakers, ventricular assist devices, and central nervous system shunts [27,28]. Among them, catheter-related infections are the major cause of morbidity and mortality among hospitalized patients, and microbial biofilms are associated with 90% of these infections. *Candida* spp. catheter-associated biofilms can lead to bloodstream infections, with an approximate incidence of one episode per 100 hospital admissions [27,29], as well as to urinary tract infections [27,30]. Up to 70% to 80% of *Candida* spp. bloodstream infections are associated with central venous catheters [27,30]. Also, *Candida* spp. endocarditis was previously considered a rare disease, but the incidence is increasing, partly because of the increased use of prosthetic intravascular devices. Likewise, biofilm formation on biotic surfaces has been reported, including both oral and vaginal tissues [31,32]. 

Among *Candida* spp., *C. albicans* is effectively the most predominant cause of invasive fungal infections and is a serious challenge for public health. However, while *C. albicans* is the fungal species most often isolated, the incidence of non-*Candida albicans Candida* species (NCACs) has recently increased [27,33,34]. In fact, more than half of the cases of *Candida* spp. infections in European countries were caused by *C. albicans*, followed by 14% for *C. glabrata*, 14% for *Candida parapsilosis*, 7% for *Candida tropicalis*, and 2% for *Candida krusei* [27,35]. A predominance of NCACs was also observed in the north of America. In Brazil, *C. albicans* accounted for 40.9% of cases, followed by *C. tropicalis* (20.9 %), *C. parapsilosis* (20.5%), and *C. glabrata* (4.9%) [27,36,37]. The change in epidemiology observed in past years could be associated with severe immunosuppression or illness, prematurity, exposure to broad-spectrum antibiotics, and older patients [27,38]. Among NCACs, *C. parapsilosis* has emerged as a significant pathogen with clinical manifestations, such as endophthalmitis, endocarditis, septic arthritis, peritonitis, and fungaemia, usually associated with invasive procedures or prosthetic devices [27,33,37,39]. A study isolated 100 strains of *C. parapsilosis* from a haemodialysis unit; 53% corresponded to *C. parapsilosis* and 47% were found to be *Candida orthopsilosis* [27]. Furthermore, candidaemia due to *C. tropicalis* has been associated with cancer in patients with leukemia or neutropenia [27,40], and *Candida dubliniensis* was frequently found in combination with other species, especially *C. albicans*. Also, it was detected a high prevalence of *C. dubliniensis* in the oral cavities of HIV-infected and AIDS patients [27,41,42]. Other species have been isolated and 400 out of 1356 isolates were identified as *C. parapsilosis sensu lato* (29.5%). This species was also isolated in Spain as the second most frequent from blood after *C. albicans* [27]. Of these 400 isolates, 364 were *C. parapsilosis sensu stricto* (90.7%), *C. orthopsilosis* (8.2%), and *Candida metapsilosis* (1.1%). The incidence of *Candida guilliermondii* and *Candida rugosa* is also increasing [37,43]. *Candida rugosa* (1.1%) has been described in the oral cavity of diabetic patients [27,44] and *Candida lusitaniae* is responsible for 1% to 2% of all candidaemias [27,44].

### 2.2. Epidemiology of Bacterial Single Biofilms

It is estimated that approximately 65% of all bacterial infections are associated with bacterial biofilms (device and non-device related) [45]. Single or mixed bacterial biofilms are correlated with a broad range of infections, from indwelling medical devices to chronic tissue infections (e.g., chronic wounds, cystic fibrosis (CF)) [46,47,48].

Although the number of anaerobic species involved in infections is lower, compared with the aerobic, and the infections are mainly formed by aerobes and anaerobes pathogens [49], the correct knowledge of the usual sites of colonization by anaerobes is helpful for the identification of the microorganisms involved and to estimate paths of invasion [50]. Indeed, the most identified are *Porphyromonas* spp., *Prevotella* spp., *Fusobacterium* spp., *Pepto-streptococcus* spp., and *Actinomyces* spp., infecting brain, spinal cord, neck, lungs, oral cavity, and upper airway. The *Bacteroides fragilis* group and *Clostridium* spp. are also isolated from patients with abdominal, gastrointestinal, or genital tract infections. *Propionibacterium acnes* has been linked to acne and endophthalmitis after cataract surgery, and *Finegoldia magna* can be frequently isolated from gynecological materials and specimens from skin and soft tissue infections [50]. 

One of the most common biofilm infections is periodontitis. In this infection, *Porphyromonas gengivalis*, *Pseudomonas aerobicus*, and *Fusobacterium nucleatum* are among the causative agents, which can also be the cause of biofilms on the mucosal surfaces in the oral cavity [51]. Other relevant infection is the colonization of teeth surfaces (tartar), which can lead to the invasion of mucosal cells, changing the flow of calcium in the epithelial cells, and the release of several toxins [52]. Eventually, osteomyelitis infections can have bacterial (e.g., *S. aureus*) or fungal origin (e.g., *C. glabrata*) [53,54], and the infections occur through the bloodstream, trauma, or foregoing infections [53].

Considering medical device infections (e.g., contact lenses, mechanical heart valves, peritoneal dialysis catheters, prosthetic joints, central venous catheters, pacemakers, urinary catheters, voice prostheses), the microorganisms can be introduced during implantation of a prosthesis or derived from a transient bacteremia. Actually, the implant tissue interface is linked to a local immunological depression of the host, which allows even the less virulent members of commensal flora to colonize the biomaterials [55]. After the insertion, the microorganisms adhere to biomaterials and grow to form a biofilm [56]. Around 40% of ventricular-assisted devices, 10% of ventricular shunts, 4% of pacemakers and defibrillators, 4% of mechanical heart valves, 2% of breast implants, and 2% of joint prostheses are affected by biofilm infections [57]. *Staphylococcus aureus* and *Staphylococcus epidermidis* are respectively at the first and second positions in staphylococcal species, followed by some emerging new pathogens, such as *Staphylococcus hominis, Staphylococcus haemolyticus, Staphylococcus capitis*, and *Staphylococcus warneri* [58,59,60]. *Staphylococcus aureus* frequently colonizes the human naris, and it is a major etiological agent of nosocomial infections [61]. *Staphylococcus epidermidis* (saprophytic of the human skin) has been alarmingly involved in outbreaks of community-acquired skin infections, progressively emerging as a main opportunistic species with the rising use of medical devices [62,63]. Moreover, *P. aeruginosa, Enterococcus faecalis* [64], and bacteria from the Enterobacteriaceae family are frequently detected on infected orthopedic implants [65]. All these microorganisms are producers or strong producers of biofilms, with distinctive matrices and implant locations [65,66,67]. For example, Enterobacteriaceae and *Enterococcus* spp. are recurrently identified after pelvis surgery [58], and *Propionibacterium* spp. (mainly *Propionibacterium acnes*) are related to infected shoulder implants [68]. Around 75% of the infections originate in these leading pathogenic species while 25% consist of over 50 species [58], an element that has relevant implications for anti-infective strategies.

Infections associated to cardiovascular implantable electronic devices are relatively infrequent, but carry a considerable risk of mortality and morbidity, demanding, in several occasions, the complete extraction of the device [69,70]. At the time of surgical implantation, there can be tissue damage, resulting in the accumulation of platelets and fibrin at the suture and on the devices. Pathogens have enhanced the aptitude to colonize these locations [15]. The microbial agents related to endocarditis differ on the time in which the infection becomes symptomatic: within 60 days from cardiac surgery, bacteria are mainly from nosocomial origin (intraoperative contamination); if the infection appears after 12 months, pathogens are typically entangled to native valve endocarditis: Viridans streptococci, *S. aureus* (leading cause of infections associated to cardiovascular implantable electronic devices [71]), *Haemophilus aphrophilus, Actinobacillus actinomycetemcomitans*, *Cardiobacterium hominis, Eikenella* spp., and *Kingella* spp. To sum up, infections occurring between 2 and 12 months are a microbial combination of the other two periods [72]. Nevertheless, there are also indications of an absence of a species shift between early and late infections [73] and a rise in species of the central nervous system, as dominant pathogens (after *S. aureus*) [71]. Gram-negative *Bacillus* spp., *Enterococcus* spp., and *Candida* spp. can also be related to endocarditis, and the origin of these can also be related to dental work [10].

Among the several microorganisms that attach to contact lenses, *E. coli, P. aeruginosa, S. aureus*, and *S. epidermidis*, but also species of *Candida* spp., *Serratia* spp., and *Proteus* spp., are among the most frequent. The adhesion varies on the water content, bacterial strain, substrate nature, electrolyte concentration, and the polymer composition. The lens storage boxes have been confirmed as a source of contamination [15]. The location and extent of biofilm formation on central venous catheters depends on the duration of catheterization, depending on factors, such as the nature of the fluid administered. In fact, Gram-positive bacteria (e.g., *S. epidermidis* and *S. aureus*) do not grow well in intravenous fluids, on contrary to Gram-negative aquatic bacteria (e.g., *P. aeruginosa, Enterobacter* spp., *Klebsiella* spp.) [10,74]. Ultimately, regarding urinary catheters, these are made of silicon or latex devices, and can have a closed or an open system for urine (more prone to contamination than the first) [75]. Urinary catheters are commonly contaminated by biofilms of *S. epidermidis, E. coli, E. faecalis, P. aeruginosa, Proteus mirabilis, Klebsiella pneumoniae*, and other Gram-negative bacteria [76].

### 2.3. Epidemiology of *Candida* spp. and Bacteria Mixed Biofilms

Humans are colonized by diverse populations of bacteria and fungi when in a healthy state and in the settings of disease, and the interactions between these microbial populations can be beneficial or detrimental to the host [77]. *Candida* spp. are the most common commensal fungus that coexist with hundreds of species of bacteria in the human body. Multiple *Candida* spp., such as *C. albicans*, *C. tropicalis*, *C. glabrata*, and *C. krusei*, have all been recovered either in combination or with other bacterial species [78,79]. Alarmingly, *Candida* spp.-associated polymicrobial infections have often resulted in high mortality and morbidity in both adults and children because of their increased dissemination behavior and the current lack of diagnostic sensitivity, especially in a biofilm mode of growth [79,80,81]. Some studies have explored the *Candida* spp.–bacterial interactions in opportunistic biofilm infections, such as those on the skin, and into systemic disease, in the lungs, in the oral cavity, in the gastrointestinal tract, and vulvovaginal. Mixed biofilms of *C. albicans* and *S. epidermidis*, *Enterococcus* spp. and *S. aureus* have been found in systemic infections [17,27,82]. In particular, *S. aureus* seems to have a certain tendency to interact with *C. albicans*, as suggested by the high frequency with which *S. aureus* is isolated from the blood of patients with candidemia. Considerably, staphylococcal species and *Candida* spp. have also been found to be associated in bloodstream infections of a neonatal population [79,81], and in infective endocarditis [79,83,84]. *Candida albicans* and *S. aureus* invasion were revealed to be clearly facilitated by *ALS3* (a *C. albicans* adhesin). *Candida albicans* hyphae (highly immunogenic feature) attracts phagocytic cells, which rapidly surround *S. aureus*, before migrating to cervical lymph nodes, and leading to systemic disease, morbidity, and mortality, which suggests synergy of the infection between these two entities [85]. Importantly, a novel strategy showed that the adhesin Als3p binds to multiple staphylococcal adhesins. The work also revealed that this is necessary for *C. albicans* to transport *S. aureus* into the tissue and cause a disseminated infection in an oral co-colonization model. These tactics accelerate the invasion of *S. aureus* through mucosal barriers, leading to systemic infection in co-colonized patients [86]. Furthermore, variances in adhesion forces between *S. aureus* and different regions of *C. albicans* hyphae (“tip”, “middle”, “head”) were quantitatively confirmed, signposting that the head region is different from the remainder of the hyphae. Significantly, properties of the hyphal head region were shown to be comparable to those of budding yeast cells [87]. Notably, the interaction between these two pathogens may be lethal to the host, by causing both candidemia and bacteremia [79,80,88]. 

In the lungs, *Candida* spp. has been reported to interact with *Burkholderia cenocepacia* in patients with CF [79], and with *Mycobacterium tuberculosis* in patients with tuberculosis [79,89]. Curiously, antagonistic interactions between *Candida* spp. and bacterial species have also been observed in the lungs, with *P. aeruginosa* killing yeast hyphae and biofilms of *C. albicans* [27,79,90]. 

In the oral environment, *Candida* spp. have been found to co-exist with multiple bacterial species, including *S. aureus*, *S. mutans* (the main bacteria found on human caries), *Streptococcus gordonii*, *E. coli*, *Klebsiella* spp., and *Pseudomonas* spp. The formation of these polymicrobial biofilms has a direct correlation with the use of dentures, with biofilms forming on the surface of these dentures or on the oral mucosa itself [22,78,79,91,92]. Some bacterial species, such as *S. gordonii*, are able to enhance the development of hyphae and the formation of biofilm by *C. albicans* when in the presence of human saliva, thereby contributing to the establishment of a polymicrobial biofilm that is hard to treat [79]. 

Further, in the gastrointestinal tract, *C. albicans* often encounters *E. faecalis* [79,93,94]. These two pathogens seem to have antagonistic interactions when in polymicrobial biofilms, much like *P. aeruginosa* and *C. albicans* in the lung environment. On contrary, *E. coli* and *C. albicans* seem to work together to form biofilms in human tissues and body fluids [79,95]. The presence of *Candida* spp. in polymicrobial biofilms in the gastrointestinal tract has been shown as particularly problematic, as the associated infections have mortality rates quite higher than those of solely bacterial polymicrobial biofilms (75% compared to 30%) [79,96,97,98,99]. Finally, in the vulvovaginal environment, antagonistic effects have been reported between *Lactobacillus* spp. (*Lactobacillus rhamnosus*, *Lactobacillus acidophilus*, *Lactobacillus plantarum*, and *Lactobacillus reuteri*) and *Candida* spp., by inhibition of both hyphal and biofilm formation by the latter [79,100]. 

## 3. *Candida*/Bacteria Mixed Biofilms: Characterization and the Problematic of the Biofilms’ Drug Resistance 

### 3.1. Mixed *Candida* spp./Bacteria Biofilms: Features, Pathogenicity, and Virulence

**Figure 1:**
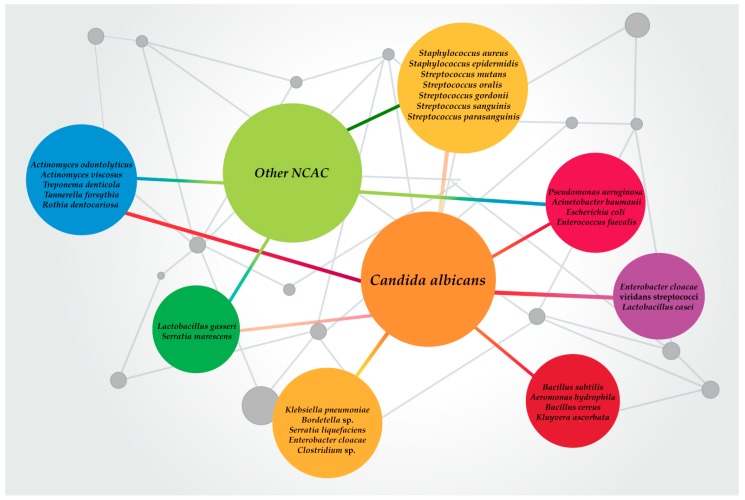
Most relevant *Candida* spp.–bacteria mixed biofilms reported and studied in the last decades.

*Candida* spp. are the most common infectious fungal species in humans. Apart from their role as the main etiology for various types of candidiasis, *Candida* spp. are also related to polymicrobial infections. In these infections, several trans-kingdom polymicrobial interactions are formed, either synergistic or antagonistic, which may support the virulence and pathogenicity of both *Candida* spp. and bacteria while distinctively impacting the pathogen–host immune response (Figure 1) [79]. 

Understanding which species—fungi and/or bacteria—controls virulence, and the associated mechanisms, especially in biofilms, offers potential for novel therapies and underpinnings for further research problems. 

It is established that different environmental conditions induce different interactions of bacteria with *Candida* spp., particularly, *C. albicans* [101]. Aerobic and anaerobic liquid co-cultures of *C. albicans* and several bacteria (*Aeromonas hydrophila*, *Bacillus cereus*, *Bacillus subtilis*, *Clostridium* spp., *Enterobacter* spp., *K. pneumoniae*, *Kluyvera ascorbate*, and *Serratia marcescens*) were used by Benadé and colleagues to study yeast–bacteria mixed cultures. *Candida albicans* growth was inhibited in the presence of bacterial growth, probably due to the presence of extracellular hydrolytic enzymes (e.g., chitinases and mannan-degrading), under aerobic conditions. Yet, this inhibition was not noticed under anaerobic conditions (no enzymes nor other compounds, such as prodigiosin from cultures of *S. marcescens*, were produced) and the growth in co-cultures was comparable to what is detected in pure cultures. A lower quantity of chitin was manufactured under anaerobic conditions, when compared to aerobic settings. Finally, the reduced production of the bacterial enzymes, prodigiosin, and mannan present in the yeast cell wall was linked to anaerobic growth and survival of *C. albicans* in the presence of bacteria [101]. Culture conditions also have influence in biofilms. Trypticase soy broth (TSB) and brain heart infusion (BHI) had higher biofilm formations and metabolic activity, and longer incubation periods with a fed-batch system and fetal bovine serum (FBS) revealed upper growth conditions in clinically isolated NCACs and *S. epidermidis* on silicone. This fact is relevant when designing or studying the mixed biofilms under *in vitro* conditions, probably being responsible for a higher or lower biomass production and, consequently, modifying the drug response [102]. As biofilms formed in silicone, some dental products can damage the oral microbiome homeostasis, inducing the mixed-species biofilm formation, allowing higher adhesion to dental prothesis. This is the case of denture adhesives (Ultra Corega Cream and Corega Strip), which increased the adhesion of *C. albicans* but not of *L. casei*. In fact, *C. albicans* biofilm formation by (single- and mixed-species) was higher on the strip adhesive. The authors did not observe any relations of synergism or antagonism between the two microorganisms [103]. 

Likewise, natural components of bacteria and fungi can condition mixed biofilm structure and functioning. For example, mannans located on the outer surface of *C. albicans* mediate *Streptococcus mutans* exoenzyme GtfB (β-glucosyltransferase) binding, so as to control *in vivo* cross-kingdom biofilm development, namely improving glucan-matrix production and regulating bacterial–fungal association. Recently, the GtfB binding properties to *C. albicans* was tested in strains defective in *O*-mannan (*pmt4*ΔΔ) or *N*-mannan outer chain (*och1*ΔΔ), and it was noticed that the binding was compromised (>3-fold reduction vs. parental strain) [64]. Moreover, the quantity of GtfB on the fungal surface was expressively cut, and the ability of *C. albicans* mutant strains to develop mixed-species biofilms with *S. mutans* was impaired (independent of hyphae or established fungal-biofilm regulators—*EFG1*, *BCR1*) [64]. As other authors have shown [104,105], the biofilm matrix stability was lower on the mutants, causing a high rate of biomass loss, which was also confirmed by *in vivo* assays [64,104,105]. The commensal protection of *S. aureus* against antimicrobials by *C. albicans* biofilm matrix has been reported. When grown together, the fungus offers a bacterial increased tolerance to antimicrobial drugs, which is secured by β-1,3-glucans secreted by the fungal cell. These polysaccharides can block the drugs’ penetration, and provide protection [104,105] through a coating of the bacteria. Notably, inhibiting β-1,3-glucans production, caspofungin indirectly sensitized the bacteria to antimicrobials [106]. In an *in vitro* model of the mixed-species biofilm *C. albicans–S. epidermidis* on polyvinyl chloride (PVC) material, the bacteria attached to the spores, pseudohyphae, and hyphae of *C. albicans*, originating a complex and dense network display. The biofilm was organized by viable and dead pathogens, and the surface of the mixed biofilms was rough, with living pathogens mostly in protrusive quotas and dead pathogens in concave aggregates [107]. Though the surface of PVC material was described as having slightly different biofilms, the formation is a dynamic process: Rapid growing in 24 h of co-culture and maximal thickness peaked at 48 h (matured at 48–72 h). Furthermore, there were noteworthy variances (*p* < 0.05) in the ratio of viable cells between the interior, middle, and outer layers [107]. Similarly, Bertolini et al. [108] evidenced that *Candida* spp.–streptococcal (*Streptococcus oralis* strain 34) mucosal biofilms exhibit distinctive structural and virulence features varying on growth conditions and hyphal morphotypes. *Streptococcus oralis* can stimulate fungal invasion and tissue damage, moisture, nutrient availability, and hyphal morphotype. Actually, the presence of commensal bacteria was shown to influence the architecture and virulence characteristics of mucosal fungal biofilms. A pioneer study in the University Hospital of Tlemcen CHU in Algeria studied the formation of mixed biofilm formation between *C. albicans* and several bacteria in peripheral venous catheters. The authors found that *C. albicans* have the potential to form mixed biofilms with *Enterobacter cloacae, Bordetella* spp., and *Serratia liquefaciens*, which were isolated from the same catheter as the yeasts. Depending on the microorganisms of the biofilms, a level of competition among bacteria and *C. albicans* was noticed that was directly associated to the composition of the medium and its pH [109].

Antagonism or synergism between *Candida* spp./bacteria mixed biofilms has also been discussed. Essentially, there is an antagonistic interaction of *S. aureus* toward *C. glabrata* during *in vitro* biofilm formation, induced by the presence of cell-free bacterial supernatant (CFBS). CFBS originated a strong decay in yeast viability and the formation of numerous lipid droplets, reactive oxygen species accumulation, as well as nuclear alterations, and DNA fragmentation signposting the initiation of an apoptotic mechanism [110]. Likewise, Martins et al. [111] explained, for the first time, the duality in *C. albicans–C. rugosa* biofilms, and suggested that *C. albicans* or *Candida* spp. can co-exist in biofilms exhibiting an apparent antagonism. Quite the reverse, earlier studies displayed synergistic interaction and increased mortality in animal models infected by dual species biofilms of *S. aureus* and *C. albicans* [112,113,114]. Zago et al. [115] clarified the dynamics of biofilm formation and the interface between *C. albicans* and methicillin-susceptible (MSSA) and -resistant *S. aureus* (MRSA). The results showed that *C. albicans*, MSSA, and MRSA can, in fact, co-exist in biofilms in an apparent synergism, with *S. aureus* cells preferentially coupled to *C. albicans* hyphal forms. Nevertheless, more studies are required, involving these and other *Candida* spp. and bacterial species.

Regarding protein receptors, Sap9 has been related to interactions amid fungal cells, and with interkingdom communication in the formation of polymicrobial biofilm communities. Compared with the parent strain, the *sap9*Δ mutant of *C. albicans* SC5314 produces smoother biofilms, with less blastopores and more hyphae. These features were stressed under flow (shear) conditions and in the presence of *S. gordonii*. Regarding dual-species biofilms (*C. albicans sap9*Δ and *S. oralis*, *Streptococcus sanguinis, Streptococcus parasanguinis*, *S. mutans*, or *E. faecalis*), all contained a higher number of entangled hyphae and bacteria bound to the substratum than the *C. albicans* wild type. Furthermore, the mutant hyphae amplified the cell surface hydrophobicity, had higher levels of the binding cell wall Als3 (~25%), and lower interaction with Eap1, which connects Sap9 in fungal cell–cell recognition [116]. The utility of intercellular adhesion A (icaA), fibrinogen binding protein (fbe), and accumulation-associated protein (aap) genes in the formation of *S. epidermidis-C. albicans* mixed-species biofilms was also explored. The thickness of *S. epidermidis* and *C. albicans* biofilms were inferior than that in the mixed biofilms. Plus, the growth speed in the mixed biofilms was greater than that in *C. albicans*, and in *S. epidermidis* at 48 h. Overall, mixed-species biofilms indicated a more complex structure and are thicker than single species biofilms of *S. epidermidis* or *C. albicans*, which can be correlated to higher expressions of the *S. epidermidis icaA, fbe*, and *aap* genes [117]. 

Prostaglandin E2 (PGE2) from *C. albicans* was proven to stimulates the growth of *S. aureus–C. albicans* in mixed biofilms, as reported by Krause et al. [118]. In fact, *C. albicans* PGE2 was determined as a central molecule stimulating growth and mixed *S. aureus/C. albicans* biofilm formation, though *C. albicans* derived farnesol, but not tyrosol, may also provide a similar stimulus but to a smaller degree [118]. 

Beforehand, it was described that microorganisms of a community, such as biofilms, secrete signaling chemical molecules to coordinate their cooperative behavior, in a phenomenon called *quorum sensing*. Kong et al. [119] confirmed that in the presence of farnesol, in biofilms (exogenously supplemented or secreted by *C. albicans*), *S. aureus* had a significantly enhanced tolerance to antimicrobials due to a broad stress response system, which can lead to upregulation of drug efflux pumps, and high resistance patterns. This work evidenced that, in mixed biofilms, *C. albicans* can improve the pathogenicity of *S. aureus*, with key therapeutic repercussions. Also, de Carvalho Dias et al. [120] stated that some soluble factors from single- and mixed-species biofilm of *C. albicans* and MSSA promote cell death and the inflammatory response. The soluble factors from mixed biofilms were the most toxic to the keratinocytes (NOK-si and HaCaT) cells. Single and mixed biofilms stimulated interleukin 6 (IL-6), nitrous oxide (NO), and tumor necrosis factor-alpha (TNF-α) production by J744A.1 macrophages [120]. 

### 3.2. Mixed *Candida* spp./Bacteria Biofilms vs. Oral Biofilms Features, Pathogenicity, and Virulence

As in oral infections, the coexistence of *Candida* spp. and bacteria in numerous other diseases is a critical issue, which questions and, in several circumstances, jeopardizes the effectiveness of the chosen therapeutics. 

This is the case of patients with CF or other respiratory disorders (ORDs). Haiko et al. [121] collected and analyzed sputum samples from 130 patients with CF and 186 patients with ORD. Respectively, nearly 70% and 44% of the sputum samples of the CF patients and patients with ORD had pathogenic bacteria, particularly *P. aeruginosa* and *S. aureus* (CF patients). No difference was noted in the coexistence of pathogenic bacteria and *Candida* spp., yet *P. aeruginosa* and *S. aureus* coexisted with *Candida* spp. more frequently in CF patients than in patients with ORD. Curiously, adult CF patients were demonstrated to have a greater rate of coexistence of any pathogenic bacteria and *Candida* spp. than the children with CF and the adult patients with ORD [121]. Different pathogens have comparable medical settings and virulence approaches in order to origin infections. Formerly, Uppuluri et al. [122] proved that active and passive immunization with Hyr1 (rHyr1p-N) protected mice against lethal candidemia. The same authors revealed that *C. albicans* Hyr1 protein can be an immunotherapeutic target for *Acinetobacter* spp. infection. Hyr1p shares its homology with cell surface proteins of the multidrug-resistant (MDR) *A. baumannii*, such as membrane protein A (OmpA), which binds to *C. albicans* Hyr1, leading to a mixed-species biofilm. Its blocking or deletion notably reduced *A. baumannii* binding to *C. albicans* hyphae, diminishing mixed biofilms’ *in vitro* formation and improving the survival of diabetic or neutropenic mice infected with *A. baumannii* bacteremia or pneumonia. 

Charles and colleagues [123] revealed that the *in vivo* decrease in anaerobic bacteria helps *C. glabrata* overgrowth (with a decrease IL-1β expression). Notably, at the same time, β-glucan treatment reestablishes the gut microbiota, mitigates colitis (increasing IL-10 production via PPARγ sensing) and, thus, *C. glabrata* elimination. During colitis development, a proliferation of *E. coli* and *E. faecalis* populations and a decline in *Lactobacillus johnsonii* and *Bacteroides thetaiotaomicron* was noted. The reduction in *L. johnsonii* was stressed by *C. glabrata* overgrowth [123]. Interactions between the gut-associated *Bacteroides fragilis* NCTC 9343, *Bacteroides vulgatus* ATCC 8482, and *C. albicans* were also explored [124]. Mostly, the yeast growth was not affected by the presence of the bacteria, but the *Bacteroides* spp. growth was expressively higher in the presence of *C. albicans*. The cell-free supernatant of 24-h-old *C. albicans* CAB 392 monocultures was able to increase the number of *Bacteroides* and the chloramphenicol sensitivity. Remarkably, the supplementation of *Bacteroides* monocultures with dead *C. albicans* CAB 392 cells (with outer cell wall mannan layers) also led to amplified bacterial concentrations. In fact, *B. vulgatus* ATCC 8482 used the mannan. The authors concluded that *C. albicans* can stimulate *Bacteroides* growth via aerobic respiration and/or antioxidant production [124]. Actually, the importance of mannans in single and mixed biofilms has previously been demonstrated [64,104]. 

Interactions between the bacteriome and mycobiome stress microbial dysbiosis in familial Crohn’s disease (CD) of northern France and Belgium have been discussed [125]. Using Ion Torrent sequencing, Hoarau and colleagues showed positive interkingdom correlations between *C. tropicalis, S. marcescens*, and *E. coli*, which were associated to CD dysbiosis. The amount of anti-*Saccharomyces cerevisiae* antibodies (ASCAa; a known CD biomarker) was related with the abundance of *C. tropicalis*. Biofilms of this species included blastopores while double- and triple-species biofilms involved hyphae. *Serratia marcescens* used fimbriae to co-aggregate or attach with *C. tropicalis–E. coli* while *E. coli* was apposed with *C. tropicalis* [125].

Bone infections (such as chronic osteomyelitis) caused by microbial biofilms are a noteworthy public health burden, with a relevant assorted morbidity and mortality. Authors have studied the pathogenesis of several species linked to this disease, performing *in vitro* and *ex vivo* assays, with several osteomyelitis pathogens in single and mixed biofilms [126]. *Staphylococcus aureus, P. aeruginosa, C. albicans*, and *S. mutans* were grown in hydroxyapatite, rat jawbone, or polystyrene wells, and in diverse media. All species produced mature biofilms within 7 days on all substrate surfaces regardless of the media. In fact, the results also showed that biofilms noticeably damaged the bone, which confirmed that osteomyelitis biofilms have the skill to directly resorb bone [126]. 

Diabetic foot ulcers (DFUs) are another major clinical problem aggravated by persistent bacterial infection. The understanding of macrophage–microbe interactions can lead to progress in targeted therapies for DFU healing. Macrophage gene expression and protein secretion have been shown to be disturbed by both microbial species as well as the human monocyte donor [127]. Indeed, *Staphylococcus simulans* and *C. albicans* instigate upregulation of genes associated with a pro-inflammatory (M1) phenotype. *Pseudomonas aeruginosa* triggers a rise in secretion of the pro-inflammatory cytokine and M1 marker tumor necrosis factor-alpha (TNFa) [127]. Similarly, the prevalence and impact of MDR microorganisms in microbial infected DFUs in north Egypt was recently studied [128]. Microbial profiles of diabetic foot patients with purulent wounds displayed a predominance of monomicrobial infections over polymicrobial infections (77.3% vs. 22.7%). A total of 24 bacterial isolates and 4 yeast isolates were identified. Strains of *C. albicans*, *A. baumanni*, *S. aureus*, and *K. pneumonia* were acknowledged, with a resistance on more than six of empirical antibiotics [128]. 

## 4. Management of *Candida* spp./Bacterial Biofilms: Is this the Impossible Mission?

Choosing the most suitable therapy to eradicate single or mixed *Candida* spp./bacterial biofilms has become one of the most actual challenging clinical goals. In the last years, several attempts have been made to select natural or synthetic new compounds with improved antimicrobial activity, or combining both, in order to increase this effect. Table 1 summarizes the most relevant ones and the following sections provide more detail of each one.

### 4.1. Oral Disease Management

Endodontic biofilms are polymicrobial communities (bacteria–fungi) surrounded by a polymeric matrix of polysaccharides, resistant to usual intracanal irrigants, antimicrobial drugs, and to the host immunity. In order to prevent and treat main oral biofilm-associated infections, the *in vitro* effectiveness of a Cu/CaOH_2_-based endodontic paste, against *S. aureus*, *P. aeruginosa*, and *C. albicans*, was evaluated. The paste expressively cut both the microbial replication time and cell growth. Biofilms experienced a fall in the number of cells and levels of released pyoverdine [129]. Quaternary ammonium amphiphiles (e.g., benzalkonium chloride, BAC), used as a preservative in topical formulations for ocular, skin, or nasal purposes, are a class of compounds with a wide range of commercial and industrial uses. BAC was shown to have a wide antimicrobial activity and minor enveloped viruses. Nonetheless, there are some safety concerns about its irritant and cytotoxic effect on epithelial cells, which demands caution in its applications. Perinelli et al. [130] synthesized BAC analogues (such as derivatives of leucine esters: C10, C12, and C14). Although the cytotoxic effect was dependent on the length of the hydrophobic chain, in general, the compounds showed a promising antimicrobial activity (against *S. aureus* and *Enterococcus* spp., *E. coli*, *P. aeruginosa*, and *C. albicans)*, as MIC values for C14-derivatives were equivalent to those of BAC [130]. Regarding therapeutic responses applying light, Pourhajibagher et al. [131] reported that an acrylic resin containing *Undaria pinnatifida*, using photo-activation with LED, has antimicrobial properties against planktonic and mixed biofilms forms of cariogenic microorganisms (*S. mutans*, *S. sanguinis*, and *Lactobacillus acidophilus*) and *C. albicans*, even at the lowest concentration. Correspondingly, photodynamic inactivation (PDI) on single- and multi-species biofilms of *C. albicans* and *S. sanguinis* showed reductions of 1.07 (single) and 0.39 log_10_ (mixed), demonstrating that PDI is a possible way to control these clinically important microorganisms [137]. In another work, Diogo et al. used photodynamic therapy (aPDT) with the Zn(II)chlorin e6 methyl ester (Zn(II)e_6_Me) activated by red light against monospecies and mixed-species biofilms of *E. faecalis* and *C. albicans*. The results proved that once activated with light for 60 or 90 s, Zn(II)e_6_Me damaged the normal microbial cell ultrastructure and removed approximately 60% of the biofilm’s biomass. Hence, these results show that aPDT might be an effective strategy for the eradication of endodontic biofilms in infected root canal systems. Further studies are, yet, needed [138].

In what concerns mouthwashes, Ardizzoni et al. [132] evaluated the antimicrobial activity of alcohol-free commercial mouthwashes with chlorhexidine digluconate (CHX), fluoride, essential oils, cetylpyridinium chloride (CC), and triclosan. *Candida albicans* and a cluster of viridans streptococci (frequently present in the oral cavity) were isolated from pharyngeal swabs and tested. The results showed that mouthwashes containing CHX and CC were the most successful in impairing biofilms and increasing the host response to *C. albicans*. Additionally, they were effective in damaging biofilm formation by viridans streptococci that cooperate with the cariogenic *S. mutans*, and ineffective against viridans streptococci that are natural competitors of *S. mutans*. On the contrary, in a mixed biofilm, the mouthwashes eradicated *S. salivarius* but failed to impair *C. albicans*’ biofilm forming ability [132]. Likewise, Tan et al. [133] showed that the combination activity of curcumin and 2-aminobenzimidazole against single- and mixed-species biofilms of *C. albicans* and *S. aureus* was curiously most potent on mixed biofilms. The antimicrobial activity of 0.2% polyhexamethilene biguanide (PHMB) to 2.5% NaOCl and 0.2% CHX in root canal models infected with *E. faecalis, C. albicans*, and *S. epidermidis* was also evaluated. PHMB reduced cell counts of all species. Both NaOCl and PHMB were efficient in eliminating *E. faecalis* and *S. epidermidis* from the mature dentin biofilm, but CHX was not satisfactory in this matter [135]. Comparably, *S. epidermidis* MFP5-5 and *S. xylosus* MFP28-3, *C. albicans* MFP8, *C. parapsilosis* MFP16-2, and *Candida famata* MFP29-1 were isolated from silicone facial prostheses by Ariani et al. [168]. In order to verify their antimicrobial activity, several agents used to clean facial prostheses were used: Antibacterial soap, essential oil-containing mouth rinse, ethanol 27%, chlorhexidine mouth rinse, and buttermilk. The results showed that antibacterial soap and buttermilk had the lowest activity. On the other side, CHX exhibited the highest reduction in colony forming units (CFUs) in 24-h, 2-week, and regrown mixed-species biofilms [168].

*Streptococcus mutans* is involved in tooth decay by the development of biofilm adhesion and caries, and the presence of *C. albicans* may exacerbate the demineralization process. The antimicrobial and anti-adhesion properties of micellar solutions of surfactants (cetylpyridinium chloride and cetyltrimethylammonium bromide and sufactin) and terpinen-4-ol (TP) (a natural plant product) were studied. All surfactants stimulated the antimicrobial activity of TP against *S. mutans*, proposing a specificity for membrane interactions that may be facilitated by surfactants [134]. Kim et al. [136] signposted that the association of topical antifungal fluconazole and povidone iodine (PI) can entirely suppress *C. albicans* oral carriage and mixed-biofilm formation without increasing the bacterial *in vivo* killing activity. PI increased the fluconazole efficacy, by disturbing the bacterial exopolysaccharide (EPS) matrix, through inhibition of α-glucan synthesis (which binds and sequesters fluconazole [172]) by *S. mutans* exoenzyme linked to the fungal surface. This study indicates that EPS inhibitors might be good anti-biofilmers to boost the killing efficacy. 

Finally, the colonization of acidogenic bacteria and fungi on denture materials is linked with DS and dental caries. An innovative mixed-species acidogenic biofilm model was recently developed to measure antimicrobial properties against single- and mixed-species biofilm of *C. albicans, Lactobacillus casei*, and *S. mutans*. The novel fluoride-releasing copolymer was constituted by methyl methacrylate (MMA) and 2-hydroxyethyl methacrylate (HEMA) with polymethyl methacrylate (PMMA). The intermicrobial interactions in mixed-species acidogenic biofilms were sensitive to fluoride (in mixed-species biofilms, cell densities were reduced around 10-fold, when compared with non-fluoride material), dropping the formation of polymicrobial biofilms [139]. 

### 4.2. Innovative Treatments of Other Diseases

Hospital-acquired infections and multidrug-resistant bacteria are a substantial hazard to any healthcare system. The *in vitro* antimicrobial features of flexible Corning® light-diffusing fiber (LDF) on ESKAPE and other relevant pathogens (*S. epidermidis, Streptococcus pyogenes, C. albicans*, and *E. coli*) were measured. The authors found that the LDF delivery of 405 nm violet-blue light had a significant antimicrobial activity towards a wide range of pathogens under diverse experimental conditions [140]. Regarding *in vivo* assays, the drug had efficacy in invasive candidiasis, aspergillosis, and pneumocystis. High bioavailability, positive drug interaction, and tolerability profile was also observed. 

During the present year, a new antibacterial and antifungal nanosystem composed of magnetic nanoparticles (MNPs) and a PBP10 peptide attached to the surface was synthesized [141]. MNPs were revealed to improve the antimicrobial activity of the phosphoinositide-binding domain of gelsolin, and control its mode of action against *S. aureus* MRSA Xen 30, *P. aeruginosa* Xen 5, and *Candida* spp., in both planktonic and biofilm forms. This effect reinforces the possibility of new treatment methods of infections [141]. 

Bacterial molecules and current drugs have also been under investigation for their antimicrobial activity. Otsuka et al. [143] demonstrated that the ZorO (type I toxin-antitoxin system), localized in the inner membrane, disturbs the plasma membrane integrity and potential when expressed in *E. coli*, also triggering the production of cytotoxic hydroxyl radicals. Exogenously added Ala-Leu-Leu-Arg-Leu peptide (ALLRL, required for ZorO toxicity) to *S. aureus*, *Bacillus subtilis*, and *C. albicans*, revealed to induce cell membrane damage and growth defect, with no effects on *E. coli*, revealing it as an attractive antimicrobial to Gram-positive bacteria and *C. albicans*. Although *E. faecalis* and *C. albicans* are common residents of the microbiome, both microorganisms are recorded by the Centers for Disease Control and Prevention, as serious global public health threats with amplified antimicrobial resistance directly related to these microorganisms have been recorded. EntV is a bacteriocin encoded by the *EntV* (*ef1097*) locus, documented for decreasing *C. albicans* virulence and biofilm formation by obstructing hyphal morphogenesis. Brown et al. indicated that the antifungal activity of the *E. faecalis* peptide EntV obliges protease cleavage only by gelatinase (GelE) and disulfide bond formation by DsbA. It was concluded that EntV, or an analogous compound, should be explored as a therapeutic alternative, alone or in combination with current drugs, against Gram-positive bacteria and *C. albicans* [144]. A diabetic class of drugs, thiazolidinediones (TZDs), was found to be successful *quorum sensing* quenchers, inhibiting biofilm formation. Prior findings confirmed this high antibiofilm effect of the TZD derivative thiazolidinedione-8 (S-8), in solution or incorporated into a sustained-release membrane (SRM-S-8) [173,174]. Analyzing the effect of SRM-S-8 on mixed *C. albicans*–*S. mutans* biofilm development, under flow conditions, indicated that the constant release of S-8 promotes enhanced penetration of the drug to deeper layers of dental polymicrobial biofilms, thus increasing the antimicrobial activity against the pathogens [145]. Rogiers et al. [151] concluded that anidulafungin (an antifungal drug) rises the antibacterial activity of tigecycline, thus acting synergistically, in polymicrobial biofilms of *C. albicans–S. aureus* on intraperitoneally implanted foreign bodies. Also, the abundance of *S. aureus* poly-β-(1,6)-N-acetylglucosamine was also cut with anidulafungin.

*Acinetobacter baumannii* is well adjusted to hospital environments. Its chronic infections endure predominantly due to its ability to form biofilms resistant to conventional drugs and to fragile host immune systems. A report disclosed the antibiofilm and antivirulence ability of the three most active flavonoids, fisetin, phloretin, and curcumin, against *A. baumannii* [146]. The antibiofilm activity was dose dependent, and curcumin had the highest activity, when compared with gallium nitrate (a biofilm inhibitor), inhibiting pellicle formation and the surface motility of *A. baumannii*. This compound also exposed antibiofilm activity against *C. albicans* and mixed cultures of *C. albicans*–*A. baumannii*. After a *Caenorhabditis elegans* infection model was treated with curcumin treatment, *A. baumannii* virulence was lowered, without cytotoxicity [146]. Later, the same authors, were able to indicate that voriconazole inhibits cross-kingdom interactions between *C. albicans* and *A. viscosus* through the ergosterol pathway [147]. They reported a higher biomass and virulence of mixed-species biofilms, when compared with the *A. viscosus* biofilm alone, and indicate voriconazole as a candidate strategy to combat root caries pathogens [147]. Eugenol showed concentration-dependent antibiofilm activity in single- and mixed-biofilms of drug-resistant strains of *C. albicans* and *S. mutans*, through multiple modes of action [148]. Importantly, in this work, the *C. albicans* strains used were resistant to fluconazole, itraconazole, ketoconazole, and amphotericin B, except *C. albicans* CAJ-01 and *C. albicans* MTCC3017, which were sensitive to fluconazole. *Streptococcus mutans* MTCC497 was resistant to ampicillin, azithromycin, ceftriaxone, and vancomycin [148]. In de Alteriis et al.’s [149] study, gH625-GCGKKKK (a derivative of the membranotropic peptide gH625) strongly inhibited the formation of mixed biofilms of clinical isolates of *C. tropicalis–S. marcescens* and *C. tropicalis*–*S. aureus* and reduced the biofilm architecture, interfering with cell adhesion and polymeric matrix, as well as eradicating the long-term polymicrobial biofilms on the silicone surface. 

Elshinawy et al. [150] evaluated chitosan (Ch-NPs), silver nanoparticles (Ag-NPs), and ozonated olive oil (O_3_-oil), both single or combined against endodontic pathogens, such as *E. faecalis*, *S. mutans*, and *C. albicans*. Ch-NPs had lower MIC and MBC values, with a higher antimicrobial activity than O_3_-oil against *E. faecalis*, *S. mutans*, and *C. albicans*. Synergism was found between O_3_-oil and Ch-NPs (FIC index ≤0.5), diminishing mature viable biofilms on a premolar *ex vivo* model (6-log reductions), with a complete anti-fibroblast adherent effect. An innovative two-layer nitric oxide-generating system (NOx) exposed 2- and 10-log fold CFU reductions [152]. NOx was effective against *S. aureus*, *P. aeruginosa, A. baumannii, E. coli*, and *Candida* spp. single- and mixed-species biofilms, including multidrug-resistant strains. This work suggests NOx as a possible new group of antimicrobial drugs with strong, broad-spectrum activity, and, importantly, with no signs of resistance development. The good efficacy of electrospun membranes of poly(lactic acid) (carrier matrix) and carvacrol (essential oil with antimicrobial activity) against *S. aureus* and *C. albicans* in single and mixed cultures was demonstrated by Scaffaro and colleagues [153]. A significant decrease of CFUs, biomass, and metabolic activity of 24- and 48-h biofilms was proven. This system might be used as a new tool for skin and wound bacterial–fungal infections [153]. 

Tyrosol does not reduce hydrolytic enzymes and acid production by *Candida* spp. and *S. mutans* but significantly reduces the metabolic activity of single biofilms of *Candida* spp. [154]. The use of this compound as a substitute antimicrobial for topical therapies still requires more studies. Similarly, carboxymethyl chitosan (CM-chitosan) might be a possible antibiofilm agent to be applied on voice prostheses. Tan and colleagues [158] performed several studies using this compound. They determined the influence of carboxymethyl chitosan on the mixed biofilm formation of *C. albicans, C. tropicalis, L. gasseri, S. salivarius, R. dentocariosa*, and *S. epidermidis*, on silicone over a long-term period. Results showed that on surfaces preserved by carboxymethyl chitosan, the biofilm was less dense and there were less coats of cells and profuse cellular debris, plus damaged and morphologically altered yeast cells. Then, they showed that it inhibits mixed fungal and damages the cells of bacterial biofilms on silicone. CM–chitosan also repressed the adhesion of fungi and bacteria (>90%) and stopped biofilm formation (~46%–70%), when it was added after biofilm initiation. However, although CM–chitosan inhibited *Candida* spp. yeast-to-hyphal transition, it was not able to inhibit the metabolic activity of biofilms [159]. In other reports, the same authors revealed it is possible to inhibit the activity of *Lactobacilli* supernatant (cell free) against fungal–bacterial multispecies biofilms on silicone. In fact, the *Lactobacilli* supernatant inhibited several features: Adhesion of mixed biofilms (efficiency >90%/90 min) and the metabolic activity of the biofilms (72.23% and 58.36%), also damaging the cells. The *Candida* spp. yeast-to-hyphal transition was equally reduced. The results showed that the *Lactobacilli* supernatant can possibly be an antibiofilm agent (single and mixed) for prostheses [161]. The same authors also described an inhibitory effect of probiotic *L. gasseri* and *L. rhamnosus* supernatants on single and mixed NCACs biofilm (*C. tropicalis, C. krusei*, and *C. parapsilosis*) [175]. Finally, another work of Tan et al. exhibited that CM–chitosan is effective as a sole agent, inhibiting both monomicrobial and polymicrobial biofilms (of *C. tropicalis* and *S. epidermidis*) in microplates and also on the silicone surface in short- and long-term periods [160]. First, CM–chitosan constrained planktonic growth and adhesion. Then, biofilm formation was also repressed (90 min or 12 h after biofilm initiation), demonstrating that this compound can possibly be used as an antibiofilm agent to limit monomicrobial and polymicrobial biofilm. 

Natural and *quorum sensing* compounds, such as marine compounds, essential oils (EOs), and extracts, have also been analyzed. Several marine bacterial exopolymers mediated green synthesis of noble metal nanoparticles (EP NPs) (derived from Eolian Islands, Italy, in the Mediterranean Sea) and have antimicrobial properties [142]. No activity was indicated for EP-gold NPs, except against *E. coli*, whereas EP-silver NPs exhibited a broad-spectrum of activity towards *S. aureus, E. coli*, *P. aeruginosa*, and *C. albicans* [142]. Budzyńska et al. [163] suggested *C. albicans–S. aureus* dual-species biofilm as an efficient target for the combination of EOs (geranium, citronella, and clove oils) and fluconazole or mupirocin. EOs of citrus species can prevent the formation of polymicrobial biofilms (*P. aeruginosa* and several pathogenic fungi). Pompia and grapefruit EOs constrained fungal growth (MIC: 50–250 mg/L), but no effect on *P. aeruginosa* growth was observed. Both citrus EOs inhibited the formation of bacterial and fungal single-species biofilms (minimum inhibitory concentration, MIC: 50 mg/L) and potentiated the activity of common antimicrobials. Finally, citrus EOs disturbed *quorum sensing* in *P. aeruginosa* and caused fast permeabilization of *C. albicans* membrane, demonstrating a possible application in the control of polymicrobial fungi–bacterial infections [163]. Combining drugs and essential oils lead to a limitation in dual-species biofilm formation and the elimination of the preformed mixed biofilm to a higher degree [162]. Soliman et at. [164] assessed the anti-*Candida* spp. activities of some medicinal plants. The ethanol extracts of *Avicennia marina* (Qurm), *Ziziphus spina*-Christi (Sidr), *Portulaca oleracea* (Baq’lah), *Fagonia indica* (Shoka’a), *Lawsania inermis* (Henna), *Salvadora persica* (Souwak), and *Asphodelus tenuifolius* (Kufer) were tested against *C. albicans* (yeasts and hypha), including cytotoxicity. The results proposed that *L. inermis* and *P. oleracea* extracts and/or their chemicals may be suited as antimicrobials against *C. albicans, S. aureus, P. aeruginosa, E. coli*, *A. baumannii*, and *Klebsiella pneumoniae*, with no associated toxicity [164]. Furthermore, it was detected that farnesol reduces the formation of single and mixed biofilms (total biomass 37%–90%; metabolic activity: 64%–96%) and cell viability (1.3–4.2 and 0.67–5.32 log_10_, respectively, for single- and mixed-species biofilms) [157,176]. Abdel-Rhman et al. [156] demonstrated that mixed-species biofilms, such as *P. aeruginosa–C. albicans*, can produce a protected environment that consents the tyrosol and farnesol (*C. albicans quorum sensing* compounds) might affect Egyptian clinical isolates of *P. aeruginosa*. Tyrosol proved to have antibacterial activity, also impeding the production of the virulence factors, hemolysin and protease, in *P. aeruginosa*, proposing that it powerfully affecta *P. aeruginosa* in mixed microbial biofilms. On the other side, farnesol faintly inhibited hemolysin production in this pathogen. Similarly, tyrosol showed inhibitory effects against single- and mixed-species biofilms formed by important oral pathogens. In fact, Arias et al. [155] evaluated single and mixed biofilms of *C. albicans* ATCC 10231, *C. glabrata* ATCC 90030, and *S. mutans* ATCC 25175 formed on acrylic resin (AR) and hydroxyapatite (HA) surfaces, in the presence of tyrosol, during 48 h. The molecule had an antibiofilm effect in single and mixed cultures (mostly at the highest concentration, 200 mmol/L), demonstrating that it can be valuable in the development of topical therapies dedicated to inhibiting biofilm-associated oral diseases.

Novel synthetic molecules are under exploration. The activity spectrum of the most active N1- and 2N-substituted 5-aryl-2-aminoimidazoles against single- and mixed-biofilms of Gram-positive and Gram-negative bacteria and *C. albicans* was assessed by Peeters et al. [165]. Molecules with substituents at both the N1 and 2N positions had high activity against mixed Gram-positive and yeast biofilms (monospecies and mixed), excluding biofilms formed by Gram-negative bacteria. The authors concluded that -aryl-2-aminoimidazoles can be used as anti-infective coatings on orthopedic implants, since, in general, the viability of bone cells was not disturbed, even inducing calcium deposition. Qu et al. [167] showed that guanylated polymethacrylates kill mixed fungal/bacterial biofilms, particularly *C. albicans*–*S. aureus*, denoting a possible use in antimicrobial lock therapy. The molecules displayed an increased efficacy, eradicating *C. albicans*–*S. aureus* mixed biofilms. Additionally, applying multiple combinations of current antimicrobial drugs, the performance was very good (99.98% of *S. aureus* and 82.2% of *C. albicans* were eliminated). When added to planktonic assays, the extracellular biofilm matrix, namely, β-1,3 glucans, offered protection to the cells, increasing the MIC of the polymethacrylates by 2- to 4-fold. The authors suggested that this mechanism might be lessened by chemical optimization of the polymer structure [167]. The antimicrobial activity of novel cellulose carbamates has also been assessed [169]. The compounds demonstrated both bactericide and fungicide *in vitro* activity. Particularly, ω-aminoethylcellulose carbamate showed high activity against *C. albicans* (IC50: 0.02 mg/mL) and *S. aureus* and IC50: 0.05 mg/mL). Besides, the antimicrobial activity and cytotoxicity was superior when p-amino-benzylamine was added, and a mixed cellulose carbamate had high biocompatibility, forming films on cotton and PES, with a strong activity against *S. aureus* and *K. pneumoniae* [169].

Finally, lock solutions have equally been under discussion for the prevention of biofilms. The current guidelines involve catheter removal, but the reinsertion can be defiant or risky. Lown et al. reported that a lock solution with micafungin, ethanol, and doxycycline inhibited *C. albicans* and mixed *C. albicans–S. aureus* biofilms. Beforehand, it was also confirmed the great *in vitro* activity of the same drugs as single agents for the prevention and treatment of *C. albicans* biofilms [177,178,179,180]. In this recent work, it was reported that a solution with 2% (v/v) ethanol, 0.01565 μg/mL micafungin, and 800 μg/mL doxycycline reduced metabolic activity (98%), with no fungal regrowth, applied once to prevent fungal biofilm formation. The solution also restrained the regrowth of *C. albicans* from mature polymicrobial biofilms, although this was not profusely bactericidal. Furthermore, when using 5% ethanol with low concentrations of micafungin and doxycycline, synergistic activity was found to prevent *C. albicans* single-biofilm formation [166]. 

## 5. Conclusions

Biofilm-associated infections require the multidisciplinary collaboration of experts of different areas of knowledge, including clinical microbiology, internal medicine, pharmacology, and basic science. To prevent microbial contamination, adhesion is a key step to avoid biofilm-associated infections. For that, prophylactic measures, such as good hygienic practices, are crucial. Although several progresses have been made to control and eradicate biofilm-related infections, new and innovative anti-biofilm approaches are still needed in order to ensure the effective management of biofilm infections.

## Figures and Tables

**Table 1:** Effective new treatments to fight *Candida* spp./bacteria mixed biofilms.

Mixed *Candida* spp./Bacteria Biofilm	Therapy	Activities	Reference(s)
*Candida albicans*, *Staphylococcus aureus*, *Pseudomonas aeruginosa*	Cu/CaOH_2_-based endodontic paste	Antimicrobial Antibiofilm	[129]
*Candida albicans*, *Staphylococcus aureus*, *Enterococcus* spp., *Escherichia coli*, *Pseudomonas aeruginosa*	Quaternary ammonium amphiphiles (derivatives of leucine esters: C10, C12 and C14)	Antimicrobial	[130]
*Streptococcus mutans, Streptococcus sanguinis*, *Lactobacillus acidophilus*, *Candida albicans*	Acrylic resin containing *U. pinnatifida*, ensuing photo-activation using LED	Antimicrobial	[131]
*Streptococcus mutans*, viridans streptococci, *Streptococcus salivarius*, *Candida albicans*	Alcohol-free commercial mouthwashes with chlorhexidine digluconate, fluoride and cetylpyridinium chloride	Antimicrobial Antibiofilm	[132]
*Candida albicans, Staphylococcus aureus*	Curcumin and 2-aminobenzimidazole	Antimicrobial Antibiofilm	[133]
*Streptococcus mutans*, *Candida albicans*	Micellar solutions of surfactants (cetylpyridinium chloride and cetyltrimethylammonium bromide and sufactin) and terpinen-4-ol (TP) (a plant natural product) was studied.	Antimicrobial	[134]
*Enterococcus faecalis*, *Candida albicans* and *Streptococcus epidermidis*	0.2% polyhexamethilene biguanide (PHMB)	Antimicrobial Antibiofilm	[135]
*Candida albicans, Streptococcus mutans*	Association of topical antifungal fluconazole and povidone iodine	Antimicrobial Antibiofilm	[136]
*Candida albicans* and *Streptococcus sanguinis*	Photodynamic inactivation (PDI)	Antimicrobial Antibiofilm	[137]
*Enterococcus faecalis* and *Candida albicans*	Photodynamic therapy (aPDT) with the Zn(II)chlorin e6 methyl ester (Zn(II)e_6_Me) activated by red light	Antimicrobial Antibiofilm	[138]
*Candida albicans, Lactobacillus casei*, and *Streptococcus mutans*	Fluoride-releasing copolymer, constituted by methyl methacrylate (MMA) and 2-hydroxyethyl methacrylate (HEMA) with polymethyl methacrylate (PMMA)	Antimicrobial Antibiofilm	[139]
ESKAPE and *Staphylococcus epidermidis, Streptococcus pyogenes, Candida albicans*, *Escherichia coli*	Corning® light-diffusing fiber (LDF)	Antimicrobial	[140]
*Staphylococcus aureus* MRSA (Xen 30), *Pseudomonas aeruginosa* (Xen 5) and *Candida* spp.	Magnetic nanoparticles and PBP10 (peptide)	Antimicrobial	[141]
*Staphylococcus aureus, Escherichia coli*, *Pseudomonas aeruginosa*, and *Candida albicans*	Marine bacterial exopolymers-Mediated green synthesis of noble metal nanoparticles	Antimicrobial	[142]
*Staphylococcus aureus*, *Bacillus subtilis*, and *Candida albicans*	Peptide derived from the ZorO *E. coli* toxin	Antimicrobial	[143]
Gram-positive bacteria and *Candida albicans*	EntV (bacteriocin)	Antibiofilm	[144]
*Candida albicans* and *Streptococcus mutans*	Derivative thiazolidinedione-8 (S-8), in solution or incorporated into a sustained-release membrane (SRM-S-8)	Antimicrobial Antibiofilm	[145]
*Candida albicans* and *Acinetobacter baumannii*	Fisetin, phloretin and curcumin (flavonoids)	Antibiofilm Antivirulence	[146]
*Candida albicans* and *Actinomyces viscosus*	Voriconazole	Inhibition of cross-kingdom interactions	[147]
*Candida albicans* and *Streptococcus mutans*	Eugenol	Antibiofilm	[148]
*Candida tropicalis-Serratia marcescens*, and *Candida tropicalis*-*Staphylococcus aureus*	gH625-GCGKKKK (derivative of the membranotropic peptide gH625)	Antiadhesion Antibiofilm	[149]
*Enterococcus faecalis*, *Streptococcus mutans*, and *Candida albicans*	Chitosan (Ch-NPs), silver Nanoparticles (Ag-NPs), ozonated olive oil (O_3_-oil), single or combined	Antiadhesion Antibiofilm	[150]
*Candida albicans-Staphylococcus aureus*	Anidulafungin	Rise of the antibacterial activity of tigecycline, (synergistic effect) Reduction of *S. aureus* poly-β-(1,6)-N-acetylglucosamine	[151]
*Staphylococcus aureus*, *Pseudomonas aeruginosa, Acinetobacter baumannii, Escherichia coli*, and *Candida* spp.	Two-layer nitric oxide-generating system (NOx)	Antimicrobial	[152]
*Staphylococcus aureus* and *Candida albicans*	Electrospun membranes of poly(lactic acid) and carvacrol	Antimicrobial Antibiofilm	[153]
*Candida* spp. and *Streptococcus mutans*	Tyrosol	Reduction of the metabolic activity	[154]
*Candida albicans* (ATCC 10231), *Candida glabrata* (ATCC 90030) and *Streptococcus mutans* (ATCC 25175)	Tyrosol	Antibiofilm	[155]
*Pseudomonas aeruginosa-Candida albicans*	Tyrosol and tyrosol + farnesol	Tyrosol: blockage of the production of hemolysin and protease in *P. aeruginosa* Farnesol: slight blockage of the production of hemolysin in *P. aeruginosa*	[156]
*Candida albicans* and *Streptococcus mutans*	Farnesol	Antibiofilm	[157]
*Candida albicans*, *Candida tropicalis, Lactobacillus gasseri, Streptococcus salivarius, Rothia dentocariosa*, and *Staphylococcus epidermidis*	Carboxymethyl chitosan	Antibiofilm Antiadhesion Inhibition of *Candida* spp. yeast-to-hyphal transition	[158,159,160]
Several fungal–bacterial multispecies	Lactobacilli supernatant	Antibiofilm Antiadhesion Antimicrobial Inhibition of *Candida* spp. yeast-to-hyphal transition Reduction of the metabolic activity	[161]
*Pseudomonas aeruginosa*, *Candida albicans, Staphylococcus aureus*	Combination geranium, citronella and clove (essential oils) and fluconazole or mupirocin.	Inhibition of fungal growth Antimicrobial Disturbance of *quorum sensing*	[162]
*Pseudomonas aeruginosa*, and *Candida albicans*	Pompia and grapefruit essential oils	Antimicrobial Antibiofilm	[163]
*Candida albicans*, *Staphylococcus aureus*, *Pseudomonas aeruginosa, Escherichia coli*, *Acinetobacter baumannii*, and *Klebsiella pneumoniae*	*Portulaca oleracea* (Baq’lah), *Lawsania inermis* (Henna) ethanol extracts	Antimicrobial	[164]
Gram-positive and *Candida albicans*	N1- and 2N-substituted 5-aryl-2-aminoimidazoles	Antiadhesion Antimicrobial	[165]
*Candida albicans*, *Staphylococcus aureus*	lock solution with micafungin, ethanol and doxycycline	Moderatly antibacterial Antibiofilm	[166]
*Candida albicans*, *Staphylococcus aureus*	Guanylated polymethacrylates with or without drug combinations	Antimicrobial Antibiofilm	[167]
*Staphylococcus epidermidis* (MFP5-5), *Staphylococcus xylosus* (MFP28-3), *Candida albicans* (MFP8), *Candida parapsilosis* (MFP16-2), *Candida famata* (MFP29-1)	Antibacterial soap, essential-oil-containing mouth rinse, ethanol 27%, chlorhexidine mouth rinse, and buttermilk	Antimicrobial	[168]
*Candida albicans*, *Staphylococcus aureus, Klebsiella pneumoniae*	Novel cellulose carbamates (e.g., ω-aminoethylcellulose carbamate) with or without p-amino-benzylamine	Antimicrobial	[169]
*Candida albicans*, *Staphylococcus aureus, Pseudomonas aeruginosa*	Extracts of *Chelidonium majus* (alkaloid: chelerythrine and chelidonine) single or in combination	Antimicrobial Antibiofilm	[170]
*Staphylococcus aureus* (6538), *Escherichia coli* (25922), *Candida albicans*	Phenolic compounds from winery waste (monomeric and tannin polyphenols)	Antimicrobial	[171]
